# Abstract of Proceedings of Buffalo Medical Association, May, 1874

**Published:** 1874-06

**Authors:** E. R. Barnes

**Affiliations:** Secretary


### ART. IV.—*Abstract of Proceedings of Buffalo Medical Association.*

Reported by E. R. Barnes, M. D., Secretary.

At a meeting of Buffalo Medical Association, held at the Medical College on Tuesday evening, May 5th, 1874, there were present: The President, Dr. White, in the chair, and Drs. Shaw, Gay, Lothrop, Gould, Rochester, Fowler and Barnes.

The reading of the minutes of the last meeting was dispensed with.

The President, Dr. White, then addressed the Society. He said that having been absent from the city at the time of the last meeting when he was elected to his present position, and having received the notice of this meeting only a few hours ago, he was quite unprepared to make an address such as he would desire to make on the occasion of his taking the chair. He had assisted in the formation of this Association, and for more than twenty-five years had taken an active part in its proceedings, and in so doing, had spent many of the pleasantest hours of his life. Here in former years were Flint, Sr., Hamilton, Hunt, Wilcox, Newman and the younger Flint. They had stood shoulder to shoulder, and together had striven to promote the cause of progress in medical knowledge. Dr. White had watched with interest the fluctuations in the prosperity and usefulness of this Association; his sympathy had always been with it, and he felt grateful now for the honor of an election for the second time to its presidency.

He proposed to offer a few suggestions how to make the meetings more interesting. It was well to appoint a gentleman to read a paper on a designated subject at a stated time, which would enable all to give some preliminary thought to the subject; and these papers should be fully discussed and criticized. It would be advantageous he thought to employ a stenographic reporter, under the direction of the Secretary. The cost would be small, and now that the Society was relieved from the payment of rent, could be easily met. The Secretary would be relieved of much labor, and could present more full and accurate reports, while the members, knowing that their remarks had been correctly recorded, would not be obliged to revise the reports as under the system hertofore in use.

Again, carefully prepared reports of cases were important, as an element of interest in the proceedings, and as indicating, and perhaps, stimulating to special study. Dr. White's advocacy of specialties was well known; he did not say one should not be a general practitioner, on the contrary, all should build on the foundation of the best attainable knowledge of general medicine. But the field had grown to be too extensive to be comprehended by any individual. It is no longer possible to know everything; something must be wisely left unknown. A physician must be content if he would know anything *well* to be profoundly ignorant of many things. He must select something for special study, and pursue it with devotion and diligence. This course will lead to success, while the attempt to do everything eventuates unavoidably in failure. Let theré be single hands for special duties. In large towns division of labor is to a certain extent desirable and necessary. The profession long since recognized opthalmology as a special department. A general practitioner cannot be an expert operator on the eye. Special attention may be given to dermatology. He had more than once, with advantage, called in the services of a friend somewhat skilled in this department. Microscopy closely allied as it is to pathology, is a most valuable department. It is often an important aid to diagnosis, prognosis and treatment to be able to have specimens examined in one's presence by an expert in this department. Pathology likewise presents an endless field for research. So also urinary and other analyses with the study of the diseases of the kidneys and urinary organs and of the rectum invite special study, and require special apparatus in practice. In the diagnosis of throat and thoracic diseases, we all have a general knowledge, but minute and exact knowledge is only attained by special and prolonged study and practice, as in the case óf Flint, Sr. Insanity and its kindred diseases, the neuroses can be well known only by the specialist. The diseases of the female organs of generation—gynecology—present a distinct field. No man in general practice can keep pace with the improvements in this department. Dr. White cited an instance in which he was called to an adjoining county by a very competent practitioner. The case was diagnosed to be one of peri-ovaritis, fluctuation was detected, and by means of the aspirator, a quantity of pus was drawn off. At last accounts this patient was recovering. This was not an ordinary case, and might be considered an extreme instance, but it illustrated the proposition which Dr. White was advocating.

Surgery might be subdivided with advantage in large towns.

Perhaps he might remark that the profession of this city are scarcely *au courant* with men of progress in thermometry in disease. Yet the thermometrical record deserves early consideration in fevers and all inflammatory diseases. The information thus attainable is of much importance. The observations should be made systematically and registered so as to present a complete thermometrical history of each case. For himself he used it as do others, but not thoroughly. In typhoid fever, diptheria, scarlet fever, etc., is is as important as is the watch to inform us as to the variations of the pulse.

Frequent meetings and full attendance promote not only the prosperity of the Association as a Society, but beget harmony among the members, rub off the asperities which collect on the surface for want of attrition. Let us meet here, look each other in the face, compare notes and adjourn, forgetting the ill feelings which have haunted our lonely hours for many days. The happiness of the members of the profession depends largely upon the comity extended by each one to his neighbor. Man is a social being, and one of the misfortunes incident to the practice of medicine consists in the isolation which attends it. No man must feel that he cannot assist in making the meetings useful. Even the youngest can contribute, and can gain confidence thereby. True, he may be criticised, and how otherwise can his progress be so well promoted. Questions should be freely asked and answered. Let a new era commence with the present year of increased zeal and increased progress. The spirit of the age is progressive; we must float with the current, or be left like drift wood or rubbish on the projecting shore.

The President having concluded his remarks, the Society proceeded to the regular order of business.

Dr. Gay moved that Dr. B. Bartow be invited to attend the meetings until he could qualify for membership. Carried.

Dr. Rochester moved that the Treasurer be authorized to honor the draughts of the Committee on rooms for $100, in favor of the building committee of the Young Men's Association in full for rent due by this Association. Carried.

Dr. Miner moved that hereafter the minutes be handed by the Secretary to the Editor of the Buffalo Medical and Surgical Journal for publication. Carried.

Reports of prevailing diseases being in order, Dr. SHAW stated that he had found typhoid and typho-malarial fevers very prevalent among children. In his practice scarlet fever had much diminished. He had seen but three cases since the last meeting.

DR. MINER corroberated the statements of Dr. Shaw. Had seen but one case of scarlet fever.

DR. ROCHESTER reported an increase of typhoid fever, and a very marked increase of the malarial fevers, some cases being distinctly remittent in character, others belonging to the class termed typho-malarial. He also found diarrœa of an unusually severe character very prevalent and difficult to control. This prevailed both among adults and children. He had met with a number of severe cases of dysentery. Jaundice also had been so prevalent as to confirm almost the observation of West, that it sometimes appeared to occur as an epidemic. It was due he thought to malarial poison and duodenal disease, giving rise to hepatic disorder. Had seen but one case of scarlet fever.

DR. GOULD mentioned scarlet fever, hooping cough, inflammation of the lungs, jaundice and obstinate diarrhœa.

These reports were coincided with by the remaining members.

DR. ROCHESTER would like to ask for more particular statements as to the prevailing jaundice.

DR. MINER had seen several of these cases, but could not always determine satisfactorily the cause. There is ordinarily duodenitis with epigastric tenderness nausea, etc. In all the cases he had seen there was no tenderness or pain, or indication of irritation of the mucous membrane. There was little if any constitutional disturbance. In one instance, perhaps, a little malaise. In these cases he could not discover evidence of obstruction from occlusion of the ductus communis choledicus. Dr. Rochester had said that the diarrhœa now prevalent is not very amenable to treatment. His own experience had been similar. Opium and astringents were of little service. Calomel and opium in small doses give the best results.

DR. WHITE gave for the diarrhœa one-fourth to one-eighth of a grain of calomel with one grain of opium—sometimes added opium suppositories. The symptoms of the cases of jaundice he had seen were the same as already reported. He thought the jaundice one of the developments of the gastric and intestinal irritation prevalent. He employed bland liquid nourishment, counter irritation, salines, such as the citrate of lithia and a mixture consisting of Rhubarb and Bi-Carbonate of soda in water, with sufficient stimulant added to preserve it. This was given in sufficient quantity to gently move the bowels every day or two. He gave quinine also.

Dr. Rochester had found tenderness not always present, in the jaundice now prevailing, and vomiting in but a single instance, but the stools were clay colored, and the urine highly tinged with the coloring matter of the bile, billerverdine. Hence we might infer duodenal irritation with enlargement of its glands and consequent obstruction to the escape of the bile through the common duct. The malarial poison in most of its manifestations, as in the fevers, gave rise to gastric and duodenal irritation or inflammation. When the stools were deep colored in jaundice, the indication was of a more serious lesion, i. e. of some organic disease of the liver which impaired the secretion of the bile. Roberts has pointed out that we may generally distinguish the obstructive from the non-obstructive variety, by the presence in the lattar of more or less bile in the stools, and of leucin and tyrosin in the urine, while in the obstructive form the stools are deficient in coloring matter, and the urine yields the bile acids and an excess of billeverdine. The analysis required to ascertain these conditions illustrates the use of specialists. Dr. R. had treated two of his patients in very different ways. In one case he had used soda, citrate of lithia, and quinine with warm fomentations, with satisfactory results. In the other case, that of an intemperate man of unfavorable appearance, with tenderness, &c., he used fomentations, a blister 6 by 6 inches, appropriate food, alkalies. The patient grew worse and had bleeding from the nose. He tried olive oil, ℨj. every three or four hours. On the second day the patient took a tablespoonful at each doze with great improvement, and then one-half a teacupful. The action here was mechanical lubricating the duodenal surface, and the orifice of the common duct. In the diarrhœa prevailing, he directed quiet, light diet, opiates by the mouth and rectum. Laxatives or cathartics even of the mildest kind, as castor oil with laudanum he had not found good.

Dr. Gay stated that he had used Esmarch's method for preventing hemorrhage three times; in a case of resection of the elbow; of necrosis of the femur; and of abduction of the great toes. In the latter case the toes were bent nearly to a right angle, in one foot underlying, in the other overlying the smaller toes. In one joint arthritic inflammation existed. Locomotion was much impeded. He resected one-half an inch of the extremity of the metatarsal bone, and the toes fell readily into position. He did not close the wound by sutures, only applying bandages. So far as he knew, this mode of relieving this deformity, was new in this city. In these cases no blood was lost. Dr. G. could not refrain from crediting Esmarch with the most magnificent contribution to the resources of surgery since the discovery of anæsthesia.

Dr. Rochester asked if there were not some objections to the use of this method? Was there not difficulty in finding the smaller arteries? If the vitality of the parts was low, might it not be destroyed by this proceedure?

Dr. Miner said it was often inapplicable. When vitality was low, its continued application, say for one hour, was dangerous. Had seen much sluggishness in the return of the circulation. It caused embarrassment in ligating the smaller vessels. In some cases there was danger of crowding deleterious substances into the circulation.

Dr. Harding asked if it produced insensibility to pain, so as to supercede anaesthesia?

Dr. Miner said it did diminish sensibility to some extent, but would not enable us to do away with anæsthesia.

On motion, the meeting adjourned.

